# Attitudes toward colorectal cancer and colonoscopy in Palestine: a questionnaire-based study

**DOI:** 10.1038/s41598-024-65653-6

**Published:** 2024-06-24

**Authors:** Mohamedraed Elshami, Mohammed Ayyad, Mohammed Alser, Ibrahim Al-Slaibi, Balqees M. Mohamad, Maram Albandak, Mohammad F. Dwikat, Shoruq A. Naji, Wejdan S. Isleem, Adela Shurrab, Bashar Yaghi, Yahya Ayyash Qabaja, Fatma K. Hamdan, Raneen R. Sweity, Remah T. Jneed, Khayria A. Assaf, Mohammed M. Hmaid, Iyas I. Awwad, Belal K. Alhabil, Marah N. Alarda, Amani S. Alsattari, Moumen S. Aboyousef, Omar A. Aljbour, Rinad AlSharif, Christy T. Giacaman, Ali Y. Alnaga, Ranin M. Abu Nemer, Nada M. Almadhoun, Sondos M. Skaik, Shurouq I. Albarqi, Bettina Bottcher, Nasser Abu-El-Noor

**Affiliations:** 1 Division of Surgical Oncology, Department of Surgery, University Hospitals Cleveland Medical Center, 11100 Euclid Avenue, Lakeside 7100, Cleveland, OH 44106 USA; 2 Ministry of Health, Gaza, Palestine; 3 Department of Internal Medicine, Rutgers New Jersey Medical School, Newark, NJ USA; 4 The United Nations Relief and Works Agency for Palestine Refugees in the Near East (UNRWA), Gaza, Palestine; 5 Almakassed Hospital, Jerusalem, Palestine; 6 Doctors Without Borders (Médecins Sans Frontières), Hebron, Palestine; 7 Faculty of Medicine, Al-Quds University, Jerusalem, Palestine; 8 Department of Internships, An-Najah National University Hospital, Nablus, Palestine; 9 Faculty of Pharmacy, Al-Azhar University of Gaza, Gaza, Palestine; 10 Faculty of Medicine, Islamic University of Gaza, Gaza, Palestine; 11 Faculty of Medicine, An-Najah National University, Nablus, Palestine; 12 Faculty of Dentistry, Arab American University, Jenin, Palestine; 13 Augusta Victoria Hospital, Jerusalem, Palestine; 14 Faculty of Allied Medical Sciences, Arab American University, Jenin, Palestine; 15 Faculty of Medicine, Al-Azhar University, Gaza, Palestine; 16 Faculty of Nursing, Islamic University of Gaza, Gaza, Palestine

**Keywords:** Colorectal cancer, Attitudes, Colonoscopy, Awareness, Patient preferences, Palestine, Cancer, Health care

## Abstract

Colorectal cancer (CRC) is a frequent cause of cancer-related mortality in the Palestinian population. This cross-sectional study was conducted from July 2019 to March 2020 and examined attitudes toward CRC and colonoscopy, as well as the interplay between both. Participants were recruited using convenience sampling from public spaces, governmental hospitals, and primary healthcare centers across 11 governorates in Palestine. Displaying a positive attitude was defined as agreeing on at least the median number of questions related to CRC (5 of 11 questions) or colonoscopy (6 of 10 questions). A total of 4623 participants were included. Most participants agreed that ‘early detection of CRC increases the possibility of more effective treatment’ (n = 4161, 89.7%). Similarly, the majority of participants agreed on ‘preferring a physician with a gender similar to the participants to perform the colonoscopy’ (n = 3738, 80.9%) and ‘willingness to have colonoscopy even if the participant had to pay for it’ (n = 3727, 80.6%). Furthermore, 3115 participants (67.4%) demonstrated positive attitudes toward CRC, while 2540 participants (55.0%) displayed similar attitudes toward colonoscopy. Participants from the West Bank and Jerusalem were more likely than those from the Gaza Strip to display positive attitudes toward colonoscopy (59.2% vs. 48.9%). Participants with positive attitudes toward CRC were more likely to also display positive attitudes toward colonoscopy and vice versa. About two thirds of study participants exhibited positive attitudes toward CRC, and 55.0% displayed positive attitudes toward colonoscopy. There was a reciprocal relationship between having positive attitudes toward CRC and colonoscopy.

## Introduction

Colorectal cancer (CRC) is the third most commonly diagnosed malignancy globally and is the second leading cause of cancer-related deaths^1^. Worldwide, over one million new cases of CRC are diagnosed annually, with death of more than 40% of those affected^2^. In Palestine, CRC is the second most prevalent malignancy following breast cancer. The incidence rate for CRC is 15.3 per 100,000 general population in the West Bank and Jerusalem and 10.2 per 100,000 general population in the Gaza Strip^3^.

Screening methods for CRC play a crucial role in reducing the burden of disease by enabling the removal of precancerous adenomas, proper patient risk stratification, and early detection of neoplastic lesions^4^. Current guidelines of the American Cancer Society recommend initiating CRC screening for people at average risk at the age of 45^5^. Specifically, it is recommended that those individuals undergo colonoscopy every 10 years or sigmoidoscopy every 5 years^5^.

In the United States, the level of awareness regarding CRC screening has been on the rise, with a screening rate reaching up to 60% in the American population. However, these rates could vary drastically among different subpopulations, often in relation to factors such as ethnicity, level of income, and education^6^. Previous studies from Palestine found that awareness levels of CRC risk factors and signs/symptoms were low with 60% of the population showing poor or fair awareness^7,8^. Interestingly, another study from Palestine looking into the role of CRC awareness in relation to attitudes toward colonoscopy showed that better awareness of CRC signs/symptoms and risk factors was associated with more positive attitudes toward colonoscopy as a screening modality^9^.

In the absence of a CRC screening program in Palestine^2^, examining attitudes toward CRC and colonoscopy becomes critical. Understanding the knowledge, perceptions, and beliefs surrounding CRC among the Palestinian population can provide valuable insights into potential barriers to early detection and prevention. In addition, healthcare providers and policymakers can develop targeted educational interventions to raise awareness and promote screening uptake by identifying misconceptions or cultural factors that may influence attitudes toward CRC screening^10–14^. Furthermore, assessing attitudes toward colonoscopy, a primary CRC screening modality, can help gauge acceptance and readiness for this procedure among Palestinians.

Governmental and non-governmental institutions, private healthcare organizations, and facilities operated by the United Nations Relief and Works Agency deliver healthcare services in Palestine. Most patients utilize healthcare services provided by governmental hospitals and primary healthcare centers at low co-payments or free of charge^3^. Of note, Palestine is divided into 16 governorates, with 11 located in the West Bank and Jerusalem and five located in the Gaza Strip^15^. To our knowledge, no studies have examined the attitudes toward CRC and colonoscopy among Palestinians and whether demonstrating positive attitudes toward CRC would translate into favorable attitudes toward colonoscopy and vice versa. Therefore, this study aimed to assess the attitudes toward CRC and colonoscopy in the Palestinian population and to compare them between the two main areas in Palestine, the West Bank and Jerusalem as well as the Gaza Strip Furthermore, it investigated how attitudes toward CRC may influence those related to colonoscopy.

## Materials and methods

### Study design and population

This cross-sectional study was conducted from July 2019 to March 2020. The study targeted healthy adult (aged ≥ 18 years) Palestinians who were residing in the West Bank, Jerusalem, or the Gaza Strip, representing approximately 62.2% of the total Palestinian population^16^. Exclusion criteria included having a profession or studying in the medical field, seeking treatment in oncology departments at the time of data collection, holding a citizenship other than Palestinian, and inability to complete the questionnaire.

### Sampling methods

The study participants were recruited using convenience sampling from public spaces, governmental hospitals, and primary healthcare centers across 11 governorates in Palestine: seven the West Bank and Jerusalem (Hebron, Nablus, Ramallah, Tulkarm, Bethlehem, Jenin, and Jerusalem) and four in the Gaza Strip (North of Gaza, Gaza, Middle Zone, and Khanyounis). Those governorates were chosen as they covered the majority of the Palestinian Population^3^. This was intended to increase the study cohort's representativeness^7,8,17^. Public spaces encompassed various locations including public transportation stations, markets, churches, mosques, parks, downtown areas, and malls.

### Data collection and measurement tool

A structured questionnaire was designed using questions from previous similar studies^18–22^. A back-to-back translation of the questionnaire was done. This involved translating the questionnaire from English to Arabic by two bilingual healthcare professionals that had expertise in clinical research and survey design. Subsequently, the translated questionnaire was independently back-translated to English by two other independent healthcare professionals, both of whom possessed bilingual proficiency and relevant expertise. Furthermore, the questionnaire underwent a thorough review to ensure content validity and accuracy of the translated version. This was carried out by five independent experts in the fields of coloproctology, public health, and gastroenterology. Additionally, the clarity of the questions in the Arabic version of the questionnaire was assessed in a pilot study (n = 25). The responses of the pilot study were not included in the final analysis. Lastly, the internal consistency of the questionnaire was assessed using Cronbach’s alpha, yielding a satisfactory value of 0.82.

The study questionnaire is provided in Supplementary File 1. It consisted of three sections. The first section covered sociodemographic factors including age, gender, marital status, employment status, educational level, monthly income, presence of any chronic illness, place of residence, knowing someone with cancer, and the site where the data were collected. The second section comprised 11 questions related to attitudes toward CRC. The questions were based on a 5-point Likert scale ranging from 1 = strongly disagree to 5 = strongly agree. The third section consisted of 10 questions using the same 5-point Likert scale and evaluated the participants' attitudes toward colonoscopy.

In terms of data collection, it was carried out in face-to-face interviews using Kobo Toolbox, a tool that is secure, user-friendly, and can be easily accessed via smartphones^23^. The data collectors were extensively trained to proficiently use Kobo Toolbox, enabling them to effectively guide participants in completing the questionnaire.

### Statistical analysis

The American Cancer Society recommends individuals with an average risk of CRC to initiate screening at the age of 45^24^. Consequently, participants were divided into two age groups based on this cutoff: 18–44 years and ≥ 45 years. Similarly, monthly income was categorized into two groups: < 1450 NIS and ≥ 1450 NIS, aligning with the minimum wage standard in Palestine, which is 1450 NIS (approximately $390)^25^.

Non-normally distributed continuous variables were summarized by computing the median and interquartile range (IQR). Baseline comparisons between participants from the Gaza Strip and those from the West Bank and Jerusalem were performed utilizing the Kruskal–Wallis test. On the other hand, categorical variables were summarized using frequencies and percentages, with comparisons performed utilizing Pearson’s Chi-Square test.

Agreeing (i.e., responding with ‘agree’ or ‘strongly agree’) on each of the attitude questions was described using frequencies and percentages, and comparisons between participants from the Gaza Strip versus the West Bank and Jerusalem were performed using Pearson’s Chi-Square test. For each response with ‘agree’ or ‘strongly agree’, participants were given one point. The total attitude score was computed and ranged from 0 to 11 for questions related to CRC and from 0 to 10 for questions related to colonoscopy. Consequently, the median score was calculated for each domain. In consistence with previous reporting^9^, displaying a positive attitude was defined as agreeing on at least the median number of questions related to CRC (5 of 11 questions) or colonoscopy (6 of 10 questions). Frequencies and percentages were utilized to describe demonstrating positive attitude toward CRC and colonoscopy, with comparisons between participants from the Gaza Strip versus the West Bank and Jerusalem performed using Pearson’s Chi-Square test. This was followed by running multivariable logistic regression analyses to identify factors associated with displaying positive attitude toward CRC or colonoscopy. The multivariable analyses adjusted for age-group, gender, employment status, education, monthly income, place of residence, marital status, having a chronic disease, knowing someone with cancer, and site of data collection. This model was determined a priori based on previous studies^7,8,17,26^.

Agreement on each of the questions related to CRC was compared between participants with or without positive attitude toward colonoscopy. In addition, multivariable logistic regression analysis was performed to examine the association between displaying positive attitude toward colonoscopy and agreeing on each CRC question. Similarly, agreement on each of the questions related to colonoscopy was compared between participants with or without positive attitude toward CRC. This was followed by performing multivariable logistic regression analysis to examine the association between displaying positive attitude toward CRC and agreeing on each question related to colonoscopy.

### Ethical approval

Prior to data collection, ethical approval was obtained from the Human Resources Development department at the Palestinian Ministry of Health and the Helsinki Committee in the Gaza Strip on 24 June 2017. A further approval was granted by the Research Ethics Committee at the Islamic University of Gaza on 26 June 2017. All participants were given a comprehensive overview explaining the study's aims, emphasizing that their participation in the study was voluntary. Each participant provided written informed consent prior to filling out the questionnaire, and data collection was carried out in a manner that ensured participant anonymity.

## Results

A total of 4877 participants completed the questionnaire out of 5254 individuals invited (response rate = 92.3%). Furthermore, 210 questionnaires were excluded due to missing data and 44 were excluded due to failing to meet the selection criteria. A total of 4623 questionnaires were included in final analysis (2700 from the West Bank and Jerusalem and 1923 from the Gaza Strip). Participants from the Gaza Strip were more likely to be of younger age, unemployed, and have lower income (Table 1).

**Table 1:** Characteristics of study participants.

Characteristic	Total (n = 4623)	Gaza strip (n = 1923)	West Bank and Jerusalem (n = 2700)	p-value
Age, median [IQR]	31.0 [24.0, 43.0]	30.0 [24.0, 40.0]	32.0 [24.0, 44.0]	< 0.001
Age group, n (%)				
18 to 44	3608 (78.1)	1579 (82.1)	2029 (75.1)	< 0.001
45 or older	1015 (21.9)	344 (17.9)	671 (24.9)	
Male gender, n (%)	1879 (40.6)	710 (36.9)	1169 (43.3)	< 0.001
Educational level, n (%)				
Secondary or below	2217 (47.9)	946 (49.2)	1271 (47.1)	0.16
Post-secondary	2406 (52.1)	977 (50.8)	1429 (52.9)	
Occupation, n (%)				
Unemployed/housewife	2067 (44.7)	1112 (57.8)	955 (35.4)	< 0.001
Employed	1898 (41.1)	563 (29.3)	1335 (49.4)	
Retired	96 (2.1)	29 (1.5)	67 (2.5)	
Student	562 (12.1)	219 (11.4)	343 (12.7)	
Monthly income ≥ 1450 NIS, n (%)	3039 (65.7)	559 (29.1)	2480 (91.9)	< 0.001
Marital status, n (%)				
Single	1414 (30.5)	548 (28.5)	866 (32.1)	0.032
Married	3067 (66.4)	1316 (68.4)	1751 (64.8)	
Divorced/widowed	142 (3.1)	59 (3.1)	83 (3.1)	
Having a chronic disease, n (%)	906 (19.6)	314 (16.3)	592 (21.9)	< 0.001
Knowing someone with cancer, n (%)	2395 (51.8)	1007 (52.4)	1388 (51.4)	0.52
Site of data collection, n (%)				
Public Spaces	1450 (31.4)	491 (25.5)	959 (35.5)	< 0.001
Hospitals	1659 (35.9)	690 (35.9)	969 (35.9)	
Primary healthcare centers	1514 (33.7)	742 (38.6)	772 (28.6)	

*n* number of participants, *IQR* interquartile range.

Most participants agreed that ‘early detection of CRC increases the possibility of more effective treatment’ (n = 4161, 89.7%) (Table 2). Conversely, a minority of participants agreed on the belief that ‘if you developed CRC, you would not feel that the therapy makes you sicker than the disease itself’ (n = 1289, 27.9%). Participants from the West Bank and Jerusalem were more likely than those from the Gaza Strip to agree on seven out of 11 questions related to CRC.

**Table 2:** Summary of the attitudes toward colorectal cancer.

Question	Total (n = 4623) n (%)	Gaza strip (n = 1923) n (%)	West Bank and Jerusalem (n = 2700) n (%)	p-value
Early detection of colorectal cancer increases the possibility of more effective treatment	4146 (89.7)	1701 (88.5)	2445 (90.6)	0.021
Early detection of colorectal cancer increases the chances of survival	4014 (86.8)	1632 (84.9)	2382 (88.2)	< 0.001
Colorectal cancer is not an infectious disease	3755 (81.2)	1497 (77.8)	2258 (83.6)	< 0.001
Taking herbs is not a cure for colorectal cancer	2264 (49.0)	912 (47.4)	1352 (50.1)	0.08
Colorectal cancer would not threaten your relationship with your (future) spouse	1759 (38.0)	678 (35.3)	1081 (40.0)	< 0.001
The problems that you would experience with colorectal cancer would not last for a long time	1753 (37.9)	654 (34.0)	1099 (40.7)	< 0.001
Your chances of getting colorectal cancer in the next few years are not high	1813 (39.2)	783 (40.7)	1030 (38.1)	0.08
The thought of colorectal cancer does not scare you	1593 (34.5)	585 (30.4)	1008 (37.3)	< 0.001
If you developed colorectal cancer, you would not feel that the therapy makes you sicker than the disease itself	1289 (27.9)	498 (25.9)	791 (29.3)	0.011
You will not get colorectal cancer sometime during your life	1471 (31.8)	651 (33.9)	820 (30.4)	0.012
If you developed colorectal cancer, you would live longer than 5 years	1442 (31.2)	717 (37.3)	725 (26.9)	< 0.001

*n* number of participants.

The majority of study participants agreed on ‘preferring a physician with a gender similar to the participants to perform the colonoscopy’ (n = 3738, 80.9%) and ‘willingness to have colonoscopy even if the participant had to pay for it’ (n = 3727, 80.6%) (Table 3). In contrast, about one third of study participants agreed that ‘if you were destined to develop CRC, you would think that having a colonoscopy would have prevented it’ (n = 1646, 35.6%). Participants from the West Bank and Jerusalem were more likely than those from the Gaza Strip to agree on six out of 10 questions related to colonoscopy.

**Table 3:** Summary of the attitudes toward colonoscopy.

Question	Total (n = 4623) n (%)	Gaza strip (n = 1923) n (%)	West Bank and Jerusalem (n = 2700) n (%)	p-value
If you had to pay for the colonoscopy, you would still do it	3727 (80.6)	1415 (73.6)	2312 (85.6)	< 0.001
You would not feel ashamed to lie on a table to have a colonoscopy	2865 (62.0)	1146 (59.6)	1719 (63.7)	0.005
You would mind not if a physician with a gender different from yours performed the colonoscopy on you	3129 (67.7)	1273 (66.2)	1856 (68.7)	0.07
You would prefer a physician with a gender similar to yours to perform the colonoscopy on you	3738 (80.9)	1556 (80.9)	2182 (80.8)	0.93
You do not have other problems in your life more important than having a colonoscopy	3139 (67.9)	1267 (65.9)	1872 (69.3)	0.013
Having a colonoscopy is not too painful	1189 (25.7)	403 (21.0)	786 (29.1)	< 0.001
Physicians doing colonoscopy are nice to people	2387 (51.6)	878 (45.7)	1509 (55.9)	< 0.001
Healthy people need to have a colonoscopy	2295 (49.6)	992 (51.6)	1303 (48.3)	0.026
If you were destined to develop colorectal cancer, you would think that having a colonoscopy would have prevented it	1646 (35.6)	664 (34.5)	982 (36.4)	0.20
Having a colonoscopy does not take too much time	2026 (43.8)	802 (41.7)	1224 (45.3)	0.014

*n* number of participants.

A total of 3115 participants (67.4%) demonstrated positive attitudes toward CRC, while 2540 participants (55.0%) displayed similar attitudes toward colonoscopy (Table 4). Participants from the West Bank and Jerusalem were more likely than those from the Gaza Strip to display positive attitudes toward colonoscopy (59.2% vs. 48.9%).

**Table 4:** Positive attitudes toward colorectal cancer and colonoscopy among study participants.

Domain	Total n (%)	Gaza strip n (%)	West Bank and Jerusalem n (%)	p-value
Colorectal cancer	3115 (67.4)	1274 (66.3)	1841 (68.2)	0.17
Colonoscopy	2540 (55.0)	941 (48.9)	1599 (59.2)	< 0.001

*n* number of participants.

On the multivariable logistic regression analysis, participants who were older than 45 years, attained higher levels of education, and earned higher monthly income had higher likelihood of displaying positive attitudes toward CRC (Table 5). On the contrary, residing in the West Bank and Jerusalem was associated with a lower likelihood of demonstrating positive attitudes toward CRC. Regarding positive attitudes toward colonoscopy, residing in the West Bank and Jerusalem, knowing someone with cancer, being married, being a student, and visiting hospitals and primary healthcare centers were all associated with a higher likelihood of displaying positive attitudes toward colonoscopy.

**Table 5:** Multivariable logistic regression analyzing factors associated with having positive attitude toward colorectal cancer and/or colonoscopy.

Factor	Colorectal cancer		Colonoscopy	
	AOR (95% CI)*	p-value	AOR (95% CI)*	p-value
Age group				
18 to 44	Ref	Ref	Ref	Ref
45 or older	1.28 (1.06–1.54)	0.009	1.08 (0.91–1.28)	0.38
Gender				
Female	Ref	Ref	Ref	Ref
Male	0.98 (0.83–1.16)	0.85	1.12 (0.96–1.31)	0.15
Educational level				
Secondary or below	Ref	Ref	Ref	Ref
Post-secondary	1.30 (1.13–1.49)	< 0.001	1.08 (0.95–1.23)	0.25
Occupation				
Unemployed/housewife	Ref	Ref	Ref	Ref
Employed	0.91 (0.76–1.09)	0.29	1.04 (0.88–1.23)	0.67
Retired	1.68 (0.96–2.96)	0.07	1.18 (0.75–1.87)	0.48
Student	0.94 (0.74–1.19)	0.62	1.40 (1.12–1.76)	0.004
Monthly income				
< 1450 NIS	Ref	Ref	Ref	Ref
≥ 1450 NIS	1.55 (1.29–1.86)	< 0.001	1.14 (0.96–1.35)	0.13
Marital status				
Single	Ref	Ref	Ref	Ref
Married	1.28 (1.09–1.51)	0.003	1.20 (1.03–1.41)	0.022
Divorced/widowed	1.02 (0.69–1.51)	0.93	1.13 (0.77–1.65)	0.53
Residency				
Gaza Strip	Ref	Ref	Ref	Ref
West Bank and Jerusalem	0.83 (0.70–0.99)	0.033	1.43 (1.22–1.67)	< 0.001
Having a chronic disease				
No	Ref	Ref	Ref	Ref
Yes	0.84 (0.70–1.00)	0.047	1.07 (0.91–1.26)	0.43
Knowing someone with cancer				
No	Ref	Ref	Ref	Ref
Yes	1.12 (0.99–1.28)	0.07	1.23 (1.09–1.39)	0.001
Site of data collection				
Public spaces	Ref	Ref	Ref	Ref
Hospitals	1.33 (1.14–1.56)	< 0.001	1.41 (1.22–1.64)	< 0.001
Primary healthcare centers	0.91 (0.77–1.07)	0.25	1.48 (1.26–1.73)	< 0.001

*AOR* adjusted odds ratio, *CI* confidence interval.

*Adjusted for age-group, gender, employment status, education, monthly income, place of residence, marital status, having a chronic disease, knowing someone with cancer, and site of data collection.

Participants showing positive attitude toward colonoscopy were more likely than those who did not to agree on eight out of 11 questions related to CRC (Table 6). In addition, participants displaying positive attitude toward CRC were more likely than those who did not to agree on nine out of 10 questions related to colonoscopy (Table 7).

**Table 6:** Summary of the attitudes toward colorectal cancer, stratified by attitude toward colonoscopy.

Question	No positive attitude (n = 2083) n (%)	Positive attitude (n = 2540) n (%)	AOR (95% CI)*	p-value
Early detection of colorectal cancer increases the possibility of more effective treatment	1823 (87.5)	2323 (91.5)	1.42 (1.17–1.72)	< 0.001
Early detection of colorectal cancer increases the chances of survival	1765 (84.7)	2249 (88.5)	1.30 (1.09–1.54)	0.003
Colorectal cancer is not an infectious disease	1571 (75.4)	2184 (86.0)	1.90 (1.63–2.21)	< 0.001
Taking herbs is not a cure for colorectal cancer	938 (45.0)	1326 (52.2)	1.33 (1.18–1.50)	< 0.001
Colorectal cancer would not threaten your relationship with your (future) spouse	689 (33.1)	1070 (42.1)	1.41 (1.24–1.59)	< 0.001
The problems that you would experience with colorectal cancer would not last for a long time	666 (32.0)	1087 (42.8)	1.54 (1.37–1.75)	< 0.001
Your chances of getting colorectal cancer in the next few years are not high	802 (38.5)	1011 (39.8)	1.11 (0.98–1.25)	0.10
The thought of colorectal cancer does not scare you	651 (31.3)	942 (37.1)	1.23 (1.08–1.40)	0.001
If you developed colorectal cancer, you would not feel that the therapy makes you sicker than the disease itself	486 (23.3)	803 (31.6)	1.52 (1.33–1.74)	< 0.001
You will not get colorectal cancer sometime during your life	672 (32.3)	799 (31.5)	1.01 (0.89–1.15)	0.88
If you developed colorectal cancer, you would live longer than 5 years	645 (31.0)	797 (31.4)	1.05 (0.92–1.20)	0.44

*n* number of participants, *AOR* adjusted odds ratio, *CI* confidence interval.

*Adjusted for age-group, gender, employment status, education, monthly income, place of residence, marital status, having a chronic disease, knowing someone with cancer, and site of data collection.

**Table 7:** Summary of the attitudes toward colonoscopy, stratified by attitude toward colorectal cancer.

Question	No positive attitude (n = 1508) n (%)	Positive attitude (n = 3115) n (%)	AOR (95% CI)*	p-value
If you had to pay for the colonoscopy, you would still do it	1160 (76.9)	2567 (82.4)	1.43 (1.22–1.67)	< 0.001
You would not feel ashamed to lie on a table to have a colonoscopy	842 (55.8)	2023 (64.9)	1.45 (1.27–1.65)	< 0.001
You would mind not if a physician with a gender different from yours performed the colonoscopy on you	931 (61.7)	2198 (70.6)	1.47 (1.29–1.68)	< 0.001
You would prefer a physician with a gender similar to yours to perform the colonoscopy on you	1207 (80.0)	2531 (81.3)	1.10 (0.93–1.29)	0.26
You do not have other problems in your life more important than having a colonoscopy	919 (60.9)	2220 (71.3)	1.58 (1.39–1.81)	< 0.001
Having a colonoscopy is not too painful	280 (18.6)	909 (29.2)	1.71 (1.47–2.00)	< 0.001
Physicians doing colonoscopy are nice to people	662 (43.9)	1725 (55.4)	1.57 (1.38–1.78)	< 0.001
Healthy people need to have a colonoscopy	712 (47.2)	1583 (50.8)	1.16 (1.02–1.32)	0.020
If you were destined to develop colorectal cancer, you would think that having a colonoscopy would have prevented it	499 (33.1)	1147 (36.8)	1.19 (1.04–1.36)	0.011
Having a colonoscopy does not take too much time	530 (35.1)	1496 (48.0)	1.65 (1.45–1.88)	< 0.001

*n* number of participants, *AOR* adjusted odds ratio, *CI* confidence interval.

*Adjusted for age-group, gender, employment status, education, monthly income, place of residence, marital status, having a chronic disease, knowing someone with cancer, and site of data collection.

## Discussion

This study examined the attitudes toward CRC and colonoscopy among Palestinians and the interplay between both. About two thirds of study participants demonstrated positive attitudes toward CRC, and more than half showed positive attitudes toward colonoscopy. Various sociodemographic factors, such as age, level of income, and education were associated with displaying positive attitudes toward CRC, colonoscopy, or both. Interestingly, there was a notable association between exhibiting positive attitudes toward CRC and displaying favorable attitudes toward colonoscopy, suggesting an interplay between them.

### Attitudes toward CRC

The findings of this study show that most study participants agreed on the belief that ‘early detection of CRC increases the possibility of more effective treatment’. This aligns with the existing literature, emphasizing the critical role of early diagnosis in improving outcomes for CRC patients^27–30^. Conversely, the low frequency of showing agreement on the belief that ‘if you developed CRC, you would not feel that the therapy makes you sicker than the disease itself’ highlights a potential barrier to acceptance and adherence to CRC treatment. This finding emphasizes the impact of beliefs related to treatment side effects, which can contribute to patient hesitancy. A previous study from Malaysia revealed that only a small minority (10.2%) of participants perceived themselves at low risk for CRC, while a significant proportion (43.3%) believed in the efficacy of early detection for better survival outcomes. Similar to our findings, the study also demonstrated that socio-demographic factors such as age, income, and family history of CRC could potentially influence the participants' attitudes toward CRC^18^. Furthermore, findings from a survey of 368 respondents in Armenia revealed good familiarity with CRC (84.0%) but poor awareness of its screening methods (22.0%)^31^. Despite this, the majority recognized the importance of regular screening in early detection and better outcomes (91.0%), with a notable proportion expressing worry about developing CRC (19.0%)^31^. It is also important to mention that only half of those concerned sought physician consultation, and a small percentage (7.0%) reported discussing CRC screening with a healthcare provider^31^. Addressing negative attitudes by acknowledging and resolving patients’ barriers, as well as improving communication between healthcare providers and patients could potentially reduce the burden of those negative attitudes toward CRC, ultimately improving patient outcomes.

### Attitudes toward colonoscopy

Most participants in this study preferred a physician with a similar gender to perform the colonoscopy, which may translate into higher likelihood of screening participation. This finding comes in concordance with previous studies. Chong et al. demonstrated a significant preference for colonoscopy being done by physicians with similar gender to that of the patients^32^. On the other hand, Khara et al. conducted a surveillance study and included 2138 American participants^33^. The authors found that most participants lacked gender preference toward their performing endoscopist. However, men were more likely than women to have higher preference for same-gender endoscopist, while women were more likely to prefer same-gender endoscopy nurses^33^. This demonstrates the importance of patient-centered care and individualizing management approaches to promote screening compliance.

Participants in this study displayed positive attitudes toward ‘willingness to have colonoscopy even if the participant had to pay for it’. This positive disposition suggests a perceived value of preventive healthcare measures, particularly colonoscopies, which are pivotal in early detection and prevention of CRC. However, it is important to take into consideration that displaying willingness to pay for CRC screening might not directly correlate with higher likelihood for undergoing colonoscopy. Additionally, Latunji et al. found that participants were very willing to pay for CRC screening (91.7%), however, the amount most individuals were willing to pay was generally low, which could act as a potential barrier to undergo screening colonoscopy, particularly in the absence of financial support or incentive^34^. In Palestine, the mean price for undergoing colonoscopy with biopsy is approximately 1200 NIS (approximately $322)^35^. This coupled with the fact that approximately 28% of employees in the West Bank and Gaza earn less than the official minimum wage (1450 NIS, approximately $390) highlights the financial challenges many Palestinians face in accessing colonoscopy services due to economic constraints^36^. Furthermore, these challenges are exacerbated by the limited availability of centers offering colonoscopy procedures in Palestine as reported by Qumseya et al.^19^, further reducing the incentive to undergo CRC screening among the Palestinian population.

Notably, there were regional disparities observed in displaying positive attitudes toward colonoscopy, with participants from the West Bank and Jerusalem exhibiting more positive attitudes than those from the Gaza Strip. This sheds light on the influence of geographical factors on healthcare perceptions. These variations may be attributed to the drastic differences in healthcare infrastructure, accessibility, or economic factors related to these regions. For instance, 60% of the Gazan population suffer from unemployment and 65% are under poverty line^37^. Furthermore, the Gazan population has a lower educational level and is of younger age compared to the West Bank and Jerusalem, which could also contribute to the discrepancies in healthcare literacy and healthcare seeking behavior between both areas^38^. In a previous study that surveyed 11,355 individuals to assess their attitudes toward CRC screening, about 14% had negative attitudes^39^. Factors such as gender, age, ethnicity, socioeconomic status, and reported symptoms significantly influenced attitudes^39^. Specifically, men, older individuals, and those from certain ethnic backgrounds (i.e., Indians) were more likely to display negative attitudes toward CRC screening^39^. These findings highlight the role of geographical, ethnic, and socioeconomic differences on the attitudes toward CRC screening. Understanding these distinctions is crucial for tailoring interventions that address specific challenges and needs in different populations.

### Interplay between positive attitudes toward CRC and colonoscopy

The findings of this study suggest that participants exhibiting positive attitudes toward colonoscopy were notably more inclined to also display positive attitudes toward CRC. This association indicates that individuals who are receptive to undergoing colonoscopy also tend to share a more positive outlook on various aspects related to CRC, possibly reflecting a good awareness and understanding of the disease. Furthermore, participants displaying positive attitudes toward CRC were more likely than their counterparts to demonstrate positive attitudes toward colonoscopy. This reciprocal relationship emphasizes that a positive disposition toward CRC, including beliefs about its prevention and treatment, corresponds with a greater likelihood of endorsing the utility and importance of colonoscopy as a screening tool. This underscores the interdependence of positive health attitudes and behaviors, particularly in the context of cancer prevention. Further studies are needed to consolidate the positive interplay between attitudes and behaviors related to both CRC and colonoscopy.

### Future directions

This study showed a positive association between attitudes toward CRC and colonoscopy. This can be the basis for further examination of the potential translation of such positive attitudes into increasing participation in screening for CRC with colonoscopy. Furthermore, since differences in demographic and patient-specific characteristics could influence behaviors toward CRC and colonoscopy, tailored interventions are needed to promote positive perspectives and preventive practices in colorectal health. Additionally, our findings indicate that there is a need for targeted interventions that address barriers related to CRC and colonoscopy. Future studies are required to investigate the determinants and implications of positive health attitudes toward CRC and colonoscopy and their contribution to actual practice of preventive measures.

### Limitations

This study has some limitations that warrant consideration when interpreting its findings. The use of convenience sampling may not yield a fully representative sample of the Palestinian population, potentially limiting the generalizability of the study results. Nevertheless, the considerably large sample size, high response rate, and diverse regional data collection may have alleviated this limitation. Additionally, the exclusion of individuals visiting oncology departments and those with medical backgrounds may have impacted the examined attitudes. However, their exclusion was meant in order to create a study cohort resembling the public in the Palestinian community. Lastly, it is important to note that the study evaluated the attitudes of participants who did not experience actual CRC symptoms and attitudes of CRC patients could be different.

## Conclusion

More than half of study participants displayed positive attitudes toward CRC and colonoscopy. Age, education, income level, and other sociodemographic characteristics influenced positive attitudes toward CRC and colonoscopy. On the other hand, several individual and service-related barriers against displaying positive attitudes toward CRC and colonoscopy were identified. This included concerns regarding treatment side effects and limited awareness of colonoscopy’s preventive role in CRC. Lastly, there was positively correlated interplay between attitudes toward CRC and colonoscopy, highlighting the importance of fostering positive attitudes for effective colorectal health initiatives.

### Supplementary Information

Supplementary Information.

## Data Availability

The dataset used during the current study is available from the corresponding author on reasonable request.
