# Supplementary material for: Attitudes toward colorectal cancer and colonoscopy in Palestine: a questionnaire-based study
Source: Sci Rep. 2024 Jun 24;14:14563. doi: 10.1038/s41598-024-65653-6 (PMC11196673; doi:10.1038/s41598-024-65653-6)
Supplement: Supplementary file 1 — Supplementary Information. [file 41598_2024_65653_MOESM1_ESM.doc]

**Colorectal Cancer Questionnaire**

Serial number: ………. Location: …………. Governorate: ……….

**A) Sociodemographic Data**

- Age: …….. years
- Gender: 🞏 Male 🞏 Female
- Marital status: 🞏 Single 🞏 Married 🞏 Divorced 🞏 Widowed
- Highest level of education: 🞏 Illiterate 🞏 Primary 🞏 Prep 🞏 Secondary

🞏 Bachelor degree 🞏 Postgraduate studies

- Occupation: 🞏 Unemployed/Housewife 🞏 Employed

🞏 Retired 🞏 Student

- Monthly income (NIS): ………………
- Do you have any chronic diseases? 🞏 No 🞏 Yes
- Have any of your family members or close friends had cancer? 🞏 No 🞏 Yes

**B) Attitudes toward colorectal cancer**

| Table (1): We are interested in your opinion on the following questions: | | | | | |
| --- | --- | --- | --- | --- | --- |
| Question | **1= Strongly Disagree** | **2= Disagree** | **3= Not Sure** | **4= Agree** | **5= Strongly agree** |
| 1. Early detection of colorectal cancer increases the possibility of more effective treatment. |  |  |  |  |  |
| 1. Early detection of colorectal cancer increases the chances of survival. |  |  |  |  |  |
| 1. Colorectal cancer is not an infectious disease. |  |  |  |  |  |
| 1. Taking herbs is not a cure for colorectal cancer. |  |  |  |  |  |
| 1. Colorectal cancer would not threaten your relationship with your (future) spouse. |  |  |  |  |  |
| 1. The problems that you would experience with colorectal cancer would not last for a long time. |  |  |  |  |  |
| 1. Your chances of getting colorectal cancer in the next few years are not high. |  |  |  |  |  |
| 1. The thought of colorectal cancer does not scare you. |  |  |  |  |  |
| 1. If you developed colorectal cancer, you would not feel that the therapy makes you sicker than the disease itself. |  |  |  |  |  |
| 1. You will not get colorectal cancer sometime during your life. |  |  |  |  |  |
| 1. If you developed colorectal cancer, you would live longer than 5 years. |  |  |  |  |  |

**C) Attitudes to Colonoscopy**

| Table (2): We are interested in your opinion on the following questions: | | | | | |
| --- | --- | --- | --- | --- | --- |
| Question | **1= Strongly Disagree** | **2= Disagree** | **3= Not Sure** | **4= Agree** | **5= Strongly agree** |
| 1. If you had to pay for the colonoscopy, you would still do it. |  |  |  |  |  |
| 1. You would not feel ashamed to lie on a table to have a colonoscopy. |  |  |  |  |  |
| 1. You would mind not if a physician with a gender different from yours performed the colonoscopy on you. |  |  |  |  |  |
| 1. You would prefer a physician with a gender similar to yours to perform the colonoscopy on you. |  |  |  |  |  |
| 1. You do not have other problems in your life more important than having a colonoscopy. |  |  |  |  |  |
| 1. Having a colonoscopy is not too painful. |  |  |  |  |  |
| 1. Physicians doing colonoscopy are nice to people. |  |  |  |  |  |
| 1. Healthy people need to have a colonoscopy. |  |  |  |  |  |
| 1. If you were destined to develop colorectal cancer, you would think that having a colonoscopy would have prevented it. |  |  |  |  |  |
| 1. Having a colonoscopy does not take too much time. |  |  |  |  |  |
